# Supplementary figures and images for: Bat sarbecovirus WIV1-CoV bears an adaptive mutation that alters spike dynamics and enhances ACE2 binding
Source: PLoS Pathog. 2025 Oct 16;21(10):e1013123. doi: 10.1371/journal.ppat.1013123 (PMC12588501; doi:10.1371/journal.ppat.1013123)

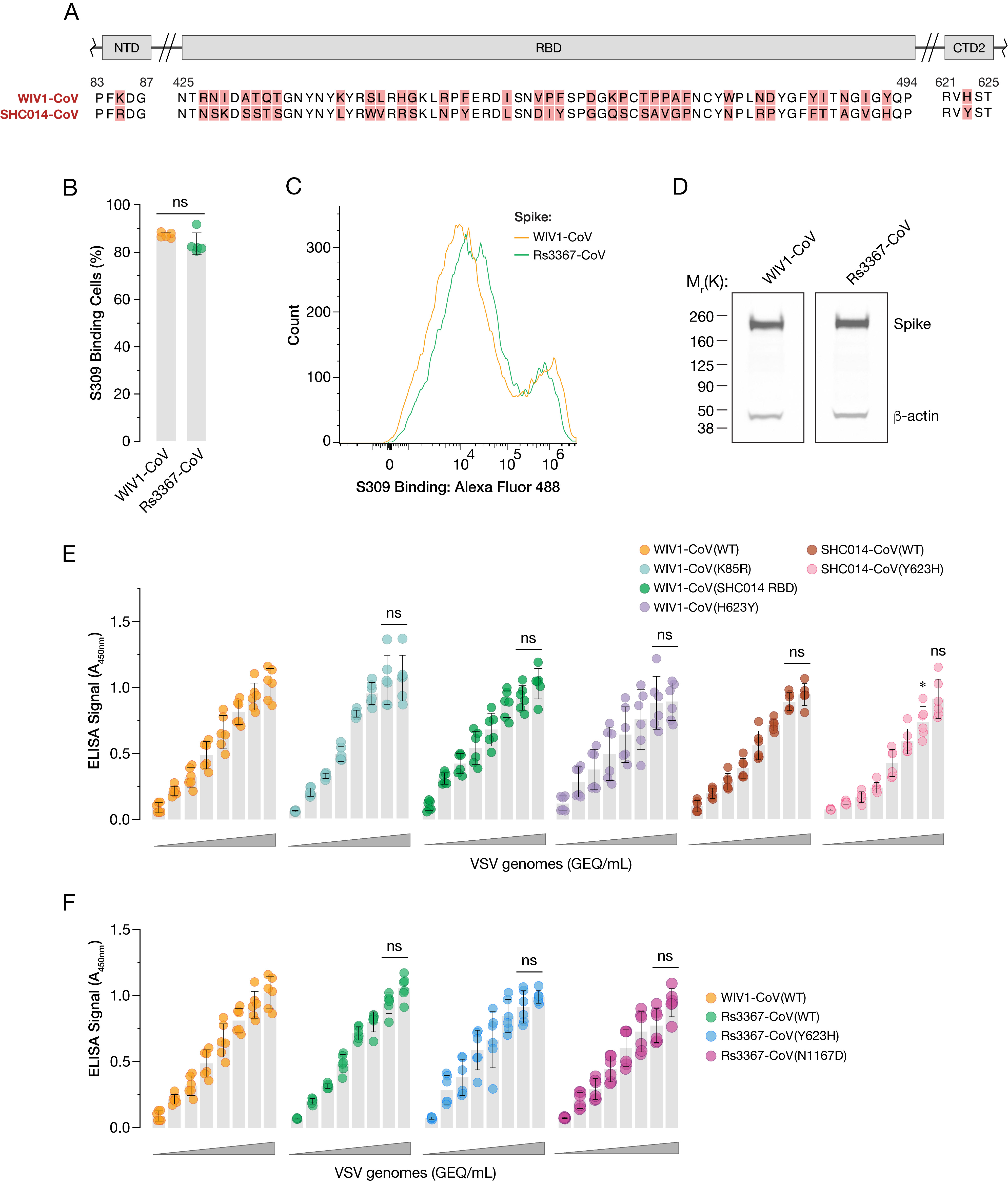

Supplement: S1 Fig — (a) Schematic representation of the amino acid differences between WIV1-CoV and SHC014-CoV in three regions of the spike proteins. Differences are highlighted in red. (b-d) 293T cells were transfected with plasmids expressing either WIV1-CoV or Rs3367-CoV spike and harvested at 24 h post-transfection. (b-c) Cells were immunostained for cell surface expression by S309, a S1-directed mAb, followed by a fluorescent secondary antibody, and analyzed using flow cytometry. Single cells were gated based on SSC-A and FSC-A and AF488-positive cells were selected by gating on transfected cells that were only stained with the fluorescent secondary antibody for background (average±SD, n = 5 from 3 independent experiments). Representative histogram shown (c). (d) Transfected cells were lysed and spike expression levels were analyzed by western blot using a S2-directed polyclonal antibody. β-actin was included as a loading control. (e-f) Genome-normalized amounts of scVSVs were diluted with 3-fold dilutions onto ELISA plates, followed by a spike-specific mAb S309 and incubation with an anti-human HRP-conjugated secondary antibody (average±SD, n = 6 from 2 independent experiments). A range of 1.0 × 10^7^ to 1.37 × 10^4^ viral GEQ was used. WIV1-CoV vs. Rs3367-CoV were compared with a non-parametric Mann-Whitney test, ns p > 0.05; ** p < 0.01; *** p < 0.001; **** p < 0.0001. (TIF) [file ppat.1013123.s001.tif]

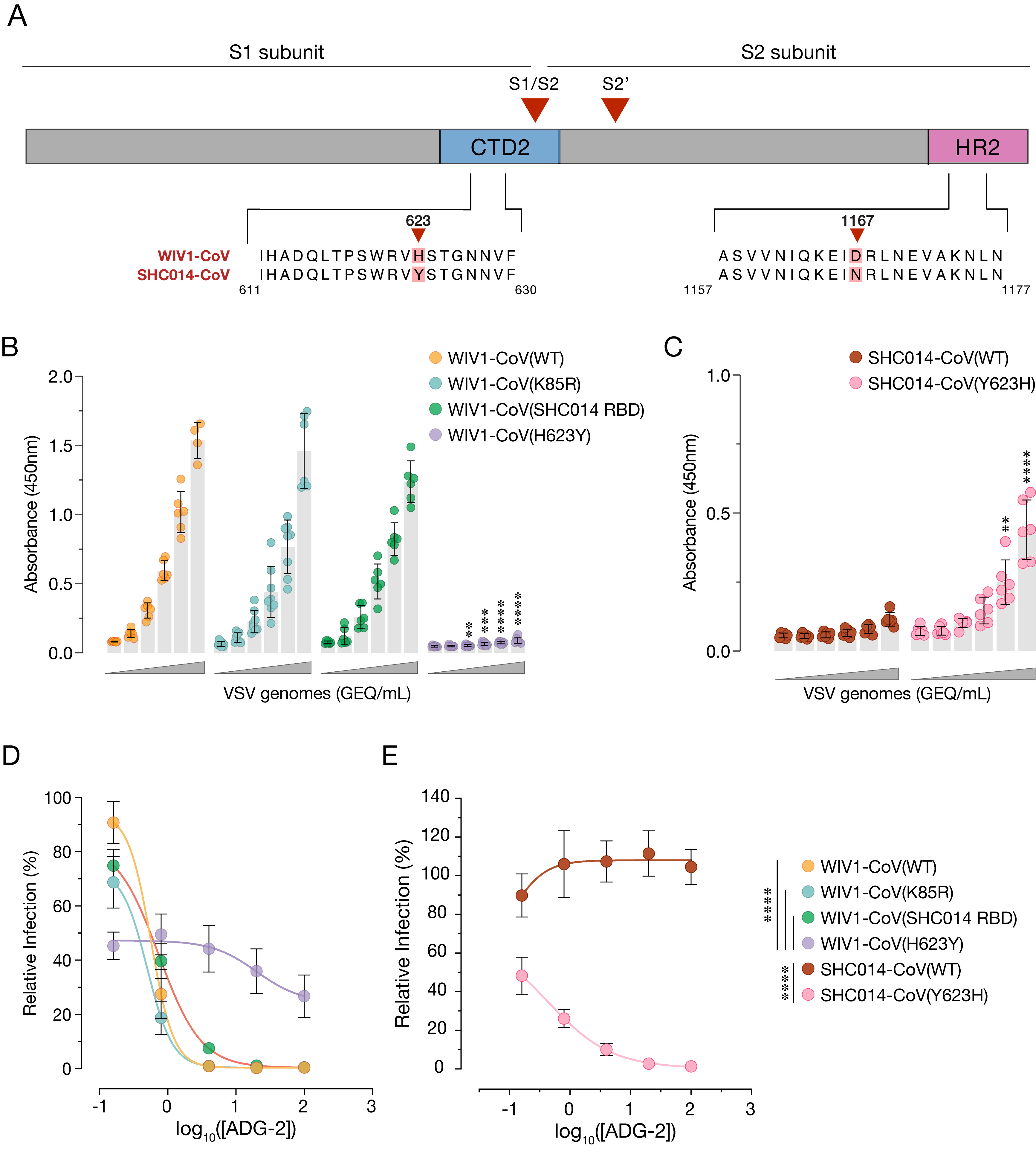

Supplement: S2 Fig — (a) Schematic representation of the CoV S protein. Locations and amino acid identities of the two point mutations that differ between Rs3367-CoV and WIV1-CoV are highlighted. (b-c) Genome-normalized amounts of scVSVs bearing spikes of WIV1-CoV (WT, K85R, SHC014 RBD, or H623Y) (b), or SHC014-CoV (WT or Y623H) (c) were diluted with 3-fold dilutions onto ELISA plates precoated with soluble HsACE2, followed by a spike-specific mAb, and incubation with an anti-human HRP-conjugated secondary antibody (average±SD, n = 6–8 from 3-4 independent experiments). A range of 9 × 10^7^ to 3.7 × 10^5^ viral GEQ was used. Groups were compared against WT with two-way ANOVA with Tukey’s correction for multiple comparisons, ns p > 0.05; ** p < 0.01; *** p < 0.001; **** p < 0.0001. (d-e) Pre-titrated amounts of scVSVs bearing spikes of WT or chimeric WIV1-CoV (d) or SHC014-CoV (e) were incubated with serial 3-fold dilutions of ADG-2 mAb, starting at 100 nM, for 1 h at 37˚C. Virus:ADG-2 mixtures were applied to monolayers of DBT-9 cells overexpressing RsACE2. At 16–18 hours post-infection, infected cells were scored by eGFP expression (average±SD, n = 9 from 3 independent experiments). Relative infectivity (%) was calculated by normalizing to no-mAb controls for each virus. AUC values were calculated for each curve, and groups were compared by one-way ANOVA with Dunnett’s post hoc test, ns p > 0.05; ** p < 0.01; *** p < 0.001; **** p < 0.0001. Only the statistically significant comparisons are shown. (TIF) [file ppat.1013123.s002.tif]

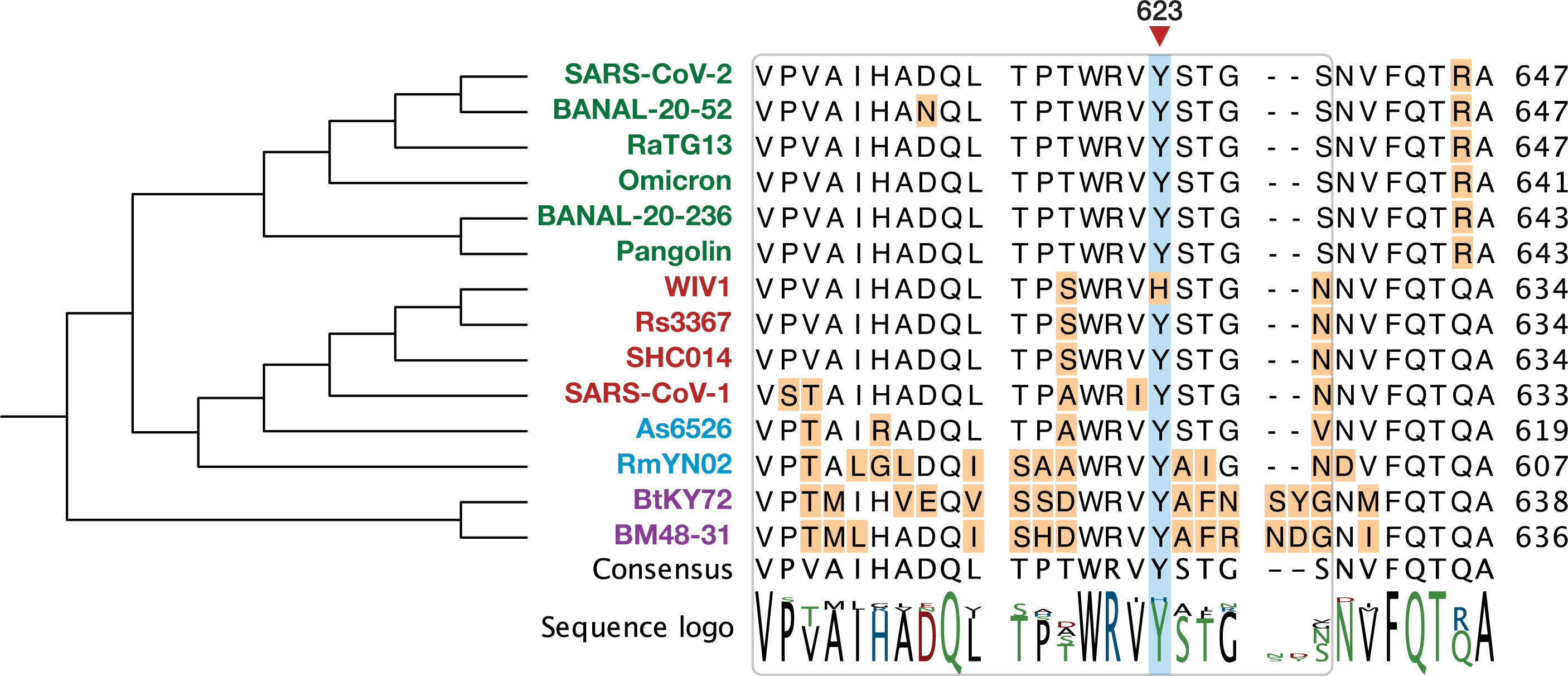

Supplement: S3 Fig — Alignment of amino acid spike sequences surrounding position 623 of the Rs3367-CoV spike protein (blue rectangle) for a panel of sarbecoviruses. Viruses are color-coded by clade; 1a: SARS-CoV-like (red), 1b: SARS-CoV-2-like (green), 2: Southeast Asian bat-origin CoV (blue), 3: non-Asian bat-origin CoV (purple). A larger sequence-based analysis using the spike of 5,944,141 sarbecoviruses was performed to assess amino acid conservation at position 623 (S1 File). (TIF) [file ppat.1013123.s003.tif]

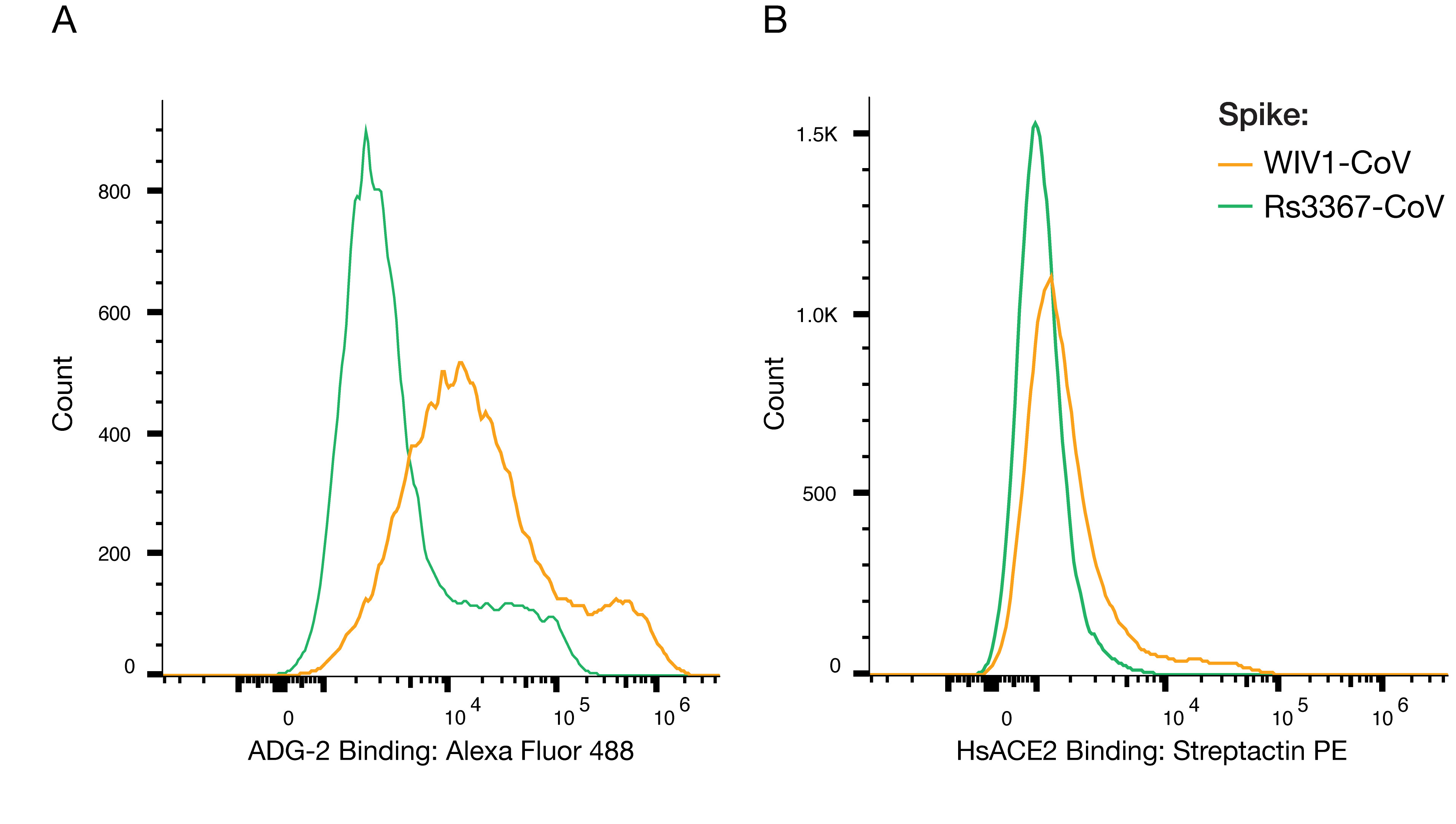

Supplement: S4 Fig — Flow cytometry analysis of 293T cells transfected with plasmids expressing either WIV1-CoV or Rs3367-CoV spikes. Cells were immunostained for cell surface expression by the S1-directed mAb ADG-2 followed by a fluorescent secondary antibody (a), or soluble HsACE2 followed by Streptactin PE (b) and analyzed using flow cytometry. Representative histograms are shown. (TIF) [file ppat.1013123.s004.tif]

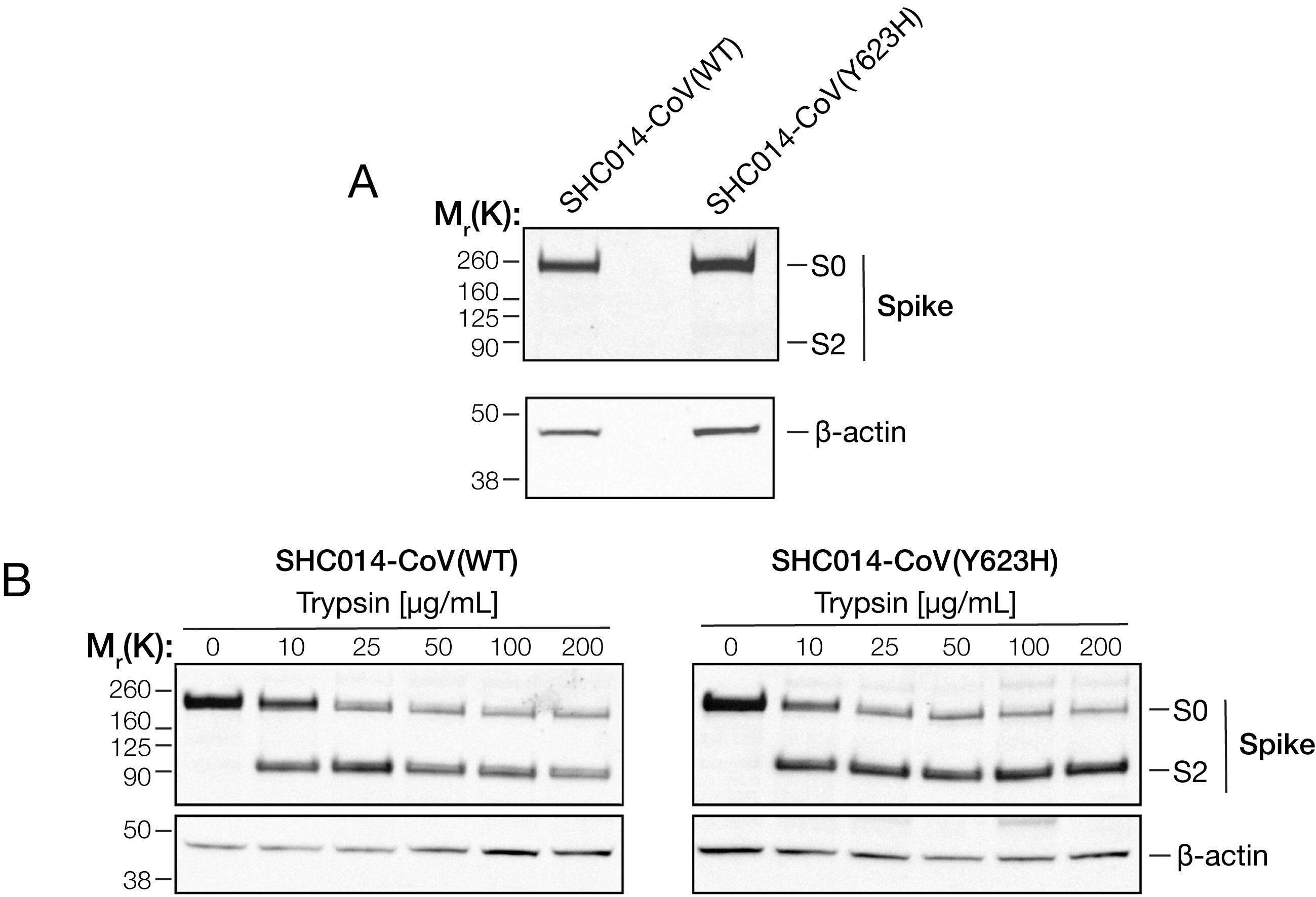

Supplement: S5 Fig — (a-b) 293T cells were transfected with plasmids to express the indicated spike proteins. At 24 h post-transfection, cells were harvested, lysed, and spike protein expression was analyzed by western blot with a CoV S2-directed polyclonal antibody. Bands corresponding to uncleaved spike (S0) or the cleaved S2 subunit (S2) are indicated based on their relative molecular weights (Mr). β-actin was included as a loading control. (b) At 24h post-transfection, spike-expressing cells were incubated with increasing amounts of trypsin for 1 h at 37˚C. (TIF) [file ppat.1013123.s005.tif]

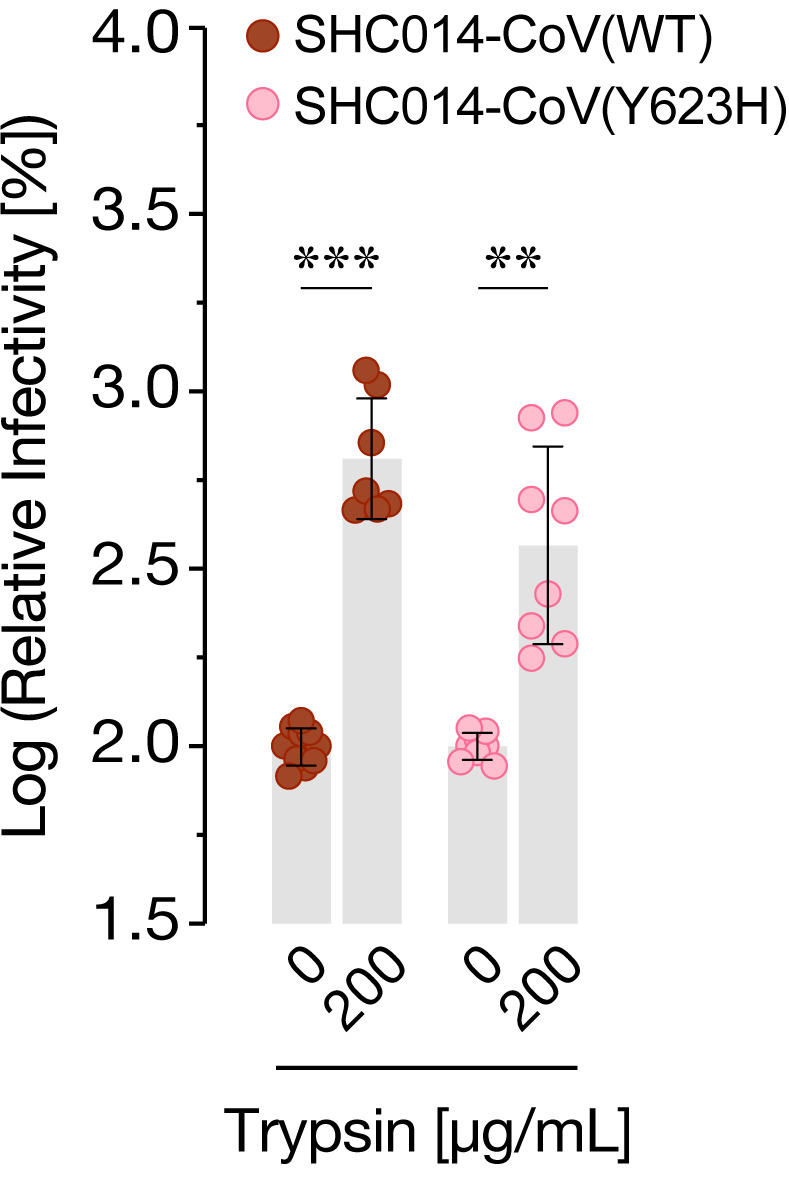

Supplement: S6 Fig — At 16–18 h post-infection, infection levels were scored by eGFP expression (average±SD. n = 6 from 3 independent experiments). Infectivity values were normalized to no-trypsin controls. Groups (no trypsin vs. trypsin treatment) were compared with unpaired t-test with Welch’s correction, ns p > 0.05; ** p < 0.01; *** p < 0.001; **** p < 0.0001. (TIF) [file ppat.1013123.s006.tif]

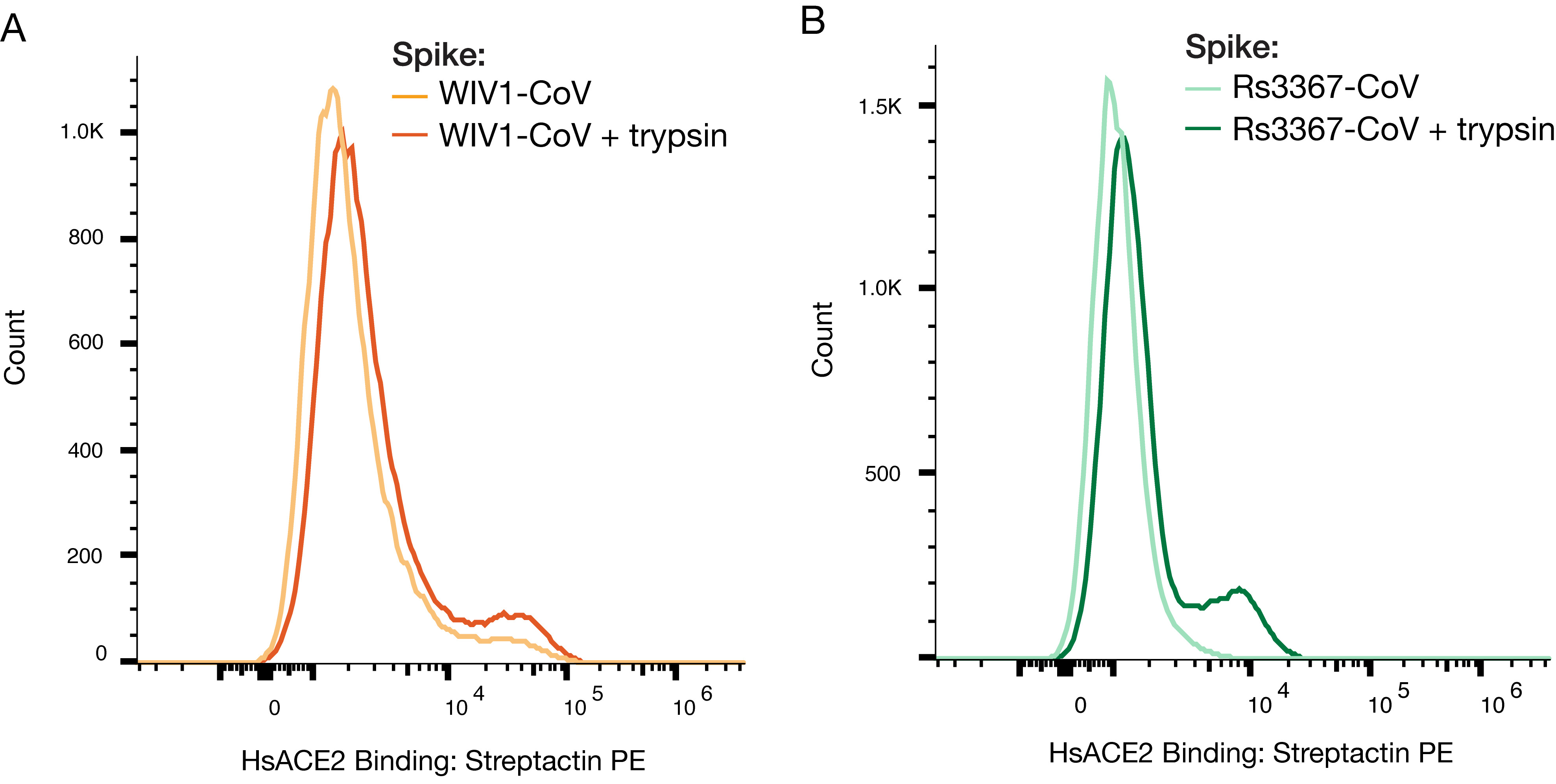

Supplement: S7 Fig — Flow cytometry analysis of 293T cells transfected with plasmids expressing the spike proteins of either (a) WIV1-CoV or (b) Rs3367-CoV. Cells were harvested and treated with 5 µg/mL of trypsin for 1 h. Cells were incubated with soluble HsACE2 followed by Streptactin PE and analyzed using flow cytometry. Representative histograms are shown. (TIF) [file ppat.1013123.s007.tif]

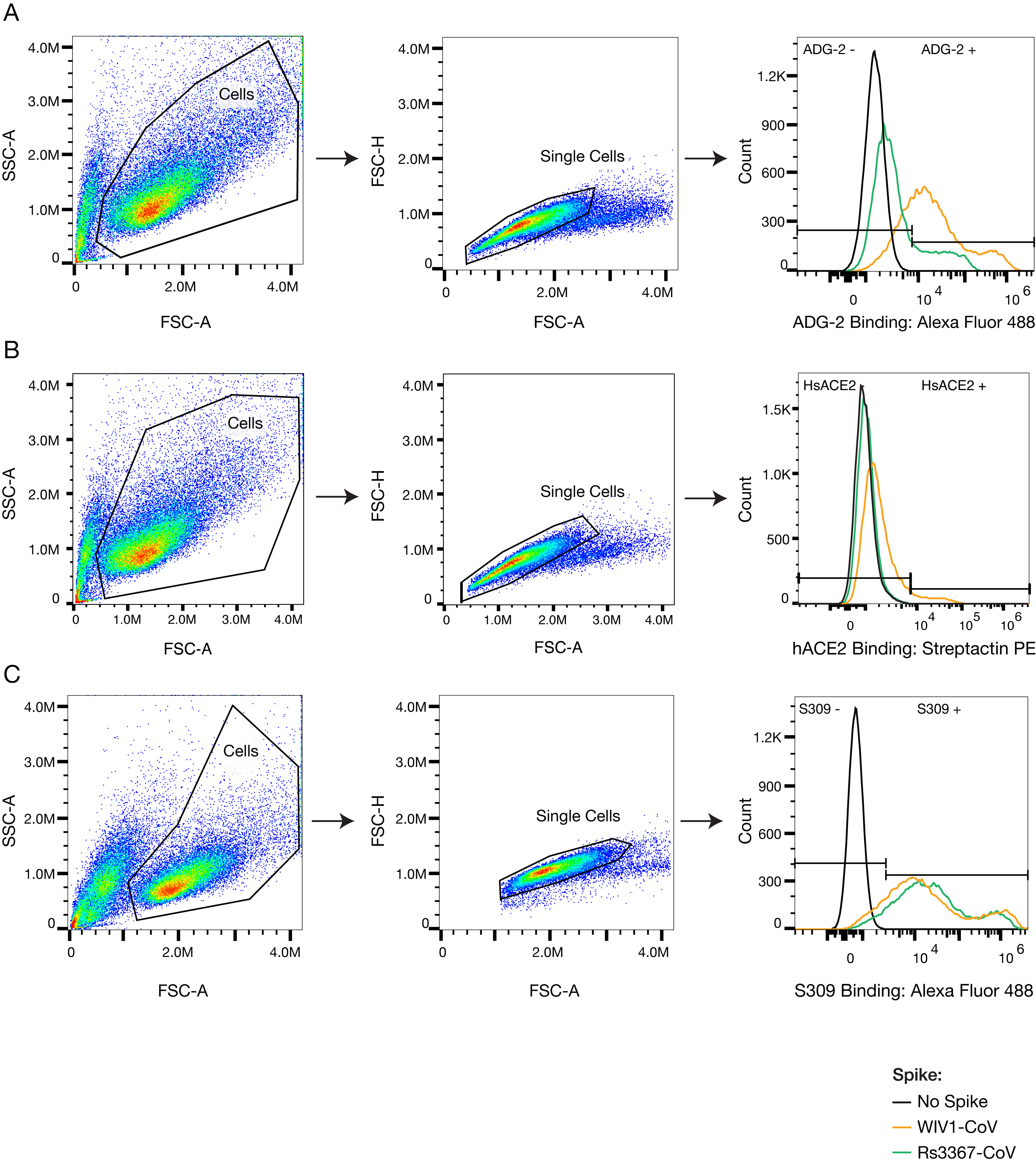

Supplement: S8 Fig — Representative gating strategy for analysis of 293T cells transfected with plasmids expressing WIV1-CoV or Rs3367-CoV spike proteins. Cells were gated based on SSC-A and FSC-A and single cells were further gated on FSC-A and FSC-H. Cells were stained with either (a) ADG-2 followed by an anti-human Alexa Fluor 488 antibody, (b) HsACE2 followed by Streptactin PE, or (c) S309 followed by anti-human Alexa Fluor 488 antibody. The gate for positive cells was set based on a negative cell population transfected with a control vector and stained as above. (TIF) [file ppat.1013123.s008.tif]

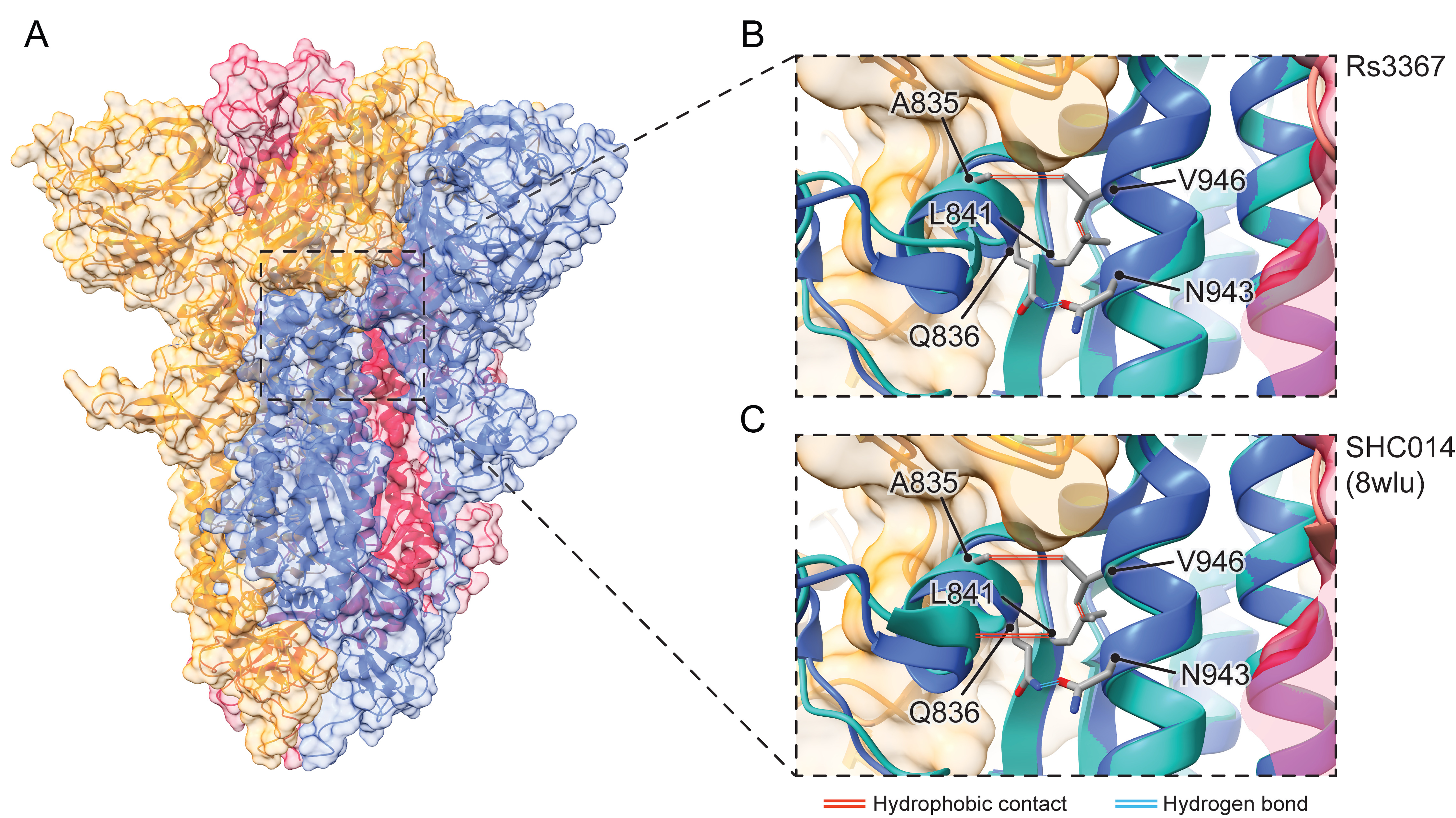

Supplement: S9 Fig — (a) Overview of an Alphafold2 model of the Rs3367-CoV spike trimer. The boxed region corresponds to the fusion-peptide proximal region (FPPR). (b-c) FPPR in the spikes of Rs3367-CoV (b) and SHC014-CoV (PDB: 8WLU) (c). Residues in Rs3367-CoV (this study; Q836, N943, V946) and SHC014-CoV (A835) [12] at which substitutions arose during rVSV rescue are highlighted, together with interacting residues. (TIF) [file ppat.1013123.s009.tif]

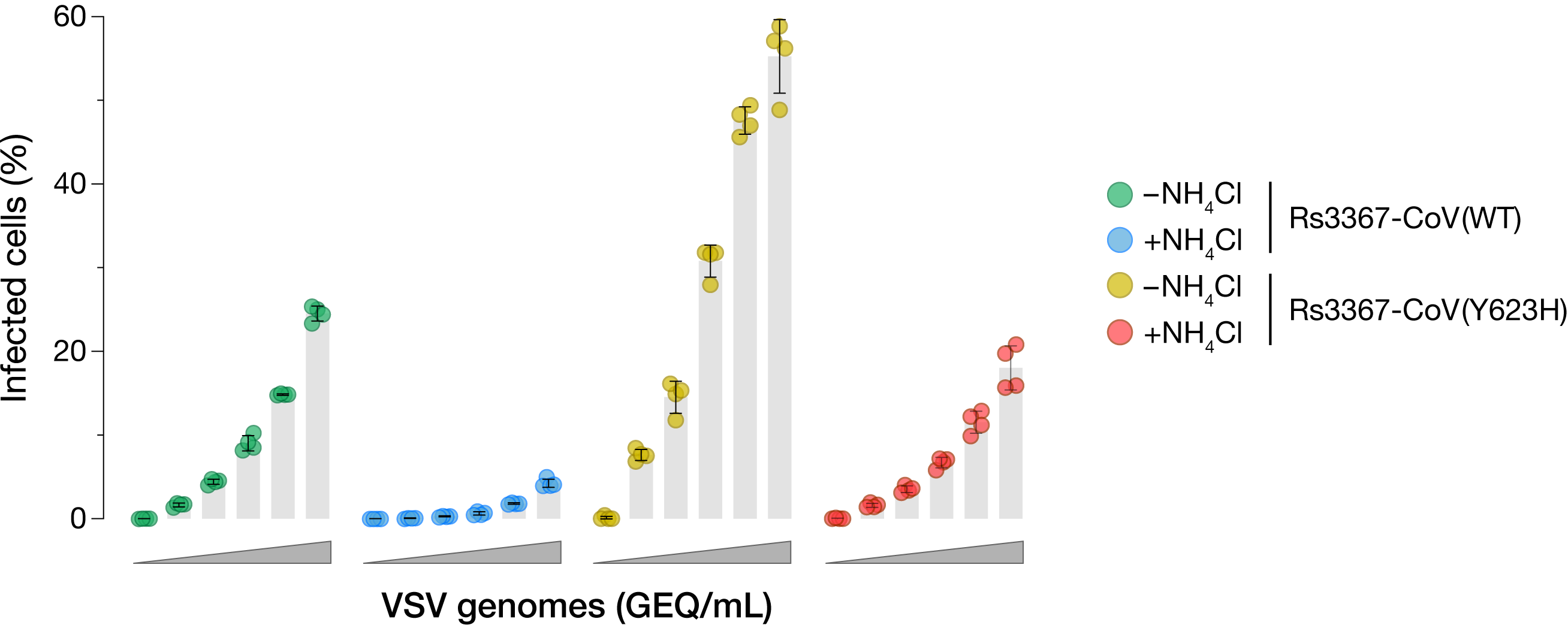

Supplement: S10 Fig — Infection was scored by eGFP expression at 16–18 hours post-infection (average±SD. n = 4 from 2 independent experiments). (TIF) [file ppat.1013123.s010.tif]
