# Supplementary material for: Bat sarbecovirus WIV1-CoV bears an adaptive mutation that alters spike dynamics and enhances ACE2 binding
Source: PLoS Pathog. 2025 Oct 16;21(10):e1013123. doi: 10.1371/journal.ppat.1013123 (PMC12588501; doi:10.1371/journal.ppat.1013123)
Supplement: Attachment — Submitted filename: Responses to reviewers_09032025_FINAL.docx [file ppat.1013123.s015.docx]

We thank the reviewers for their helpful input. Please see below for a point-by-point response.

**Reviewer #1: Major**

While the Y623H change markedly increases SHC014-CoV S protein-driven entry, entry activity is not restored to the same level measured for WIV-1 S protein. Therefore, it is important to analyze whether introducing NTD/RBD of WIV-1 into SHC014-CoV S increases entry efficiency.

This manuscript focuses on the identification of an amino acid difference in the ‘630 loop’ as a determinative change that likely influenced the evolution of WIV1-CoV from a bat precursor virus related to Rs3367-CoV. Mapping all of the sequences that influence the intrinsic difference in entry efficiency between the spikes of SHC014 and WIV1-CoV—two distinct viruses that differ substantially in amino acid sequence, is not the primary goal of this work. Rather, we use the SHC014:WIV1 analysis to illustrate the remarkable effect of a single amino acid change in the 630 loop in the background of the many other sequence differences in the NTD and RBD.

Further, Qiao and Wang (J Virol, 2024) have extensively examined and reported on the SHC014-WIV1 difference. Thus, we feel that a more detailed analysis of the SHC014-WIV1 difference is both tangential to our study and somewhat redundant with the Qiao and Wang publication. Here we focus on the evolution of WIV1-CoV from an Rs3367-CoV–like virus.

Expression of all chimeras (figure 1) in transfected cells or, preferably, incorporation into particles needs to be determined in order to allow for solid conclusions.

The results shown in figure 3 must be bolstered by data on S protein incorporation into particles.

We include new data demonstrating the similar levels of spike incorporation in all of the chimeras investigated in Figures 1 and 3 (**S1E–F Fig.**).

The conclusion that Y623H is a cell culture adaptation must be bolstered by functional data, considering that the mutation might also have been acquired during circulation in bats. Thus, it should be analyzed whether rVSV- Rs3367-S acquires Y623H or related changes upon passaging in cell culture.

We note that we are not arguing that Y623H was acquired during cell culture passage. Although this could well have occurred, there is no way to infer this from the available data. Rather, we conclude that Y623H was selected during cell culture passage. This conclusion does not presuppose that Y623H arose *de novo* in cell culture; it may well have existed in the viral quasispecies in the bat sample and then been enriched during passage.

Nevertheless, we take the reviewer’s point that it would be interesting to see if Y623H or similar substitutions might arise during rVSV-Rs3367-CoV S rescue. Accordingly, we carried out two independent rescue experiments from a VSV genome clone encoding the WT Rs3367-CoV spike. Both experiments yielded replicating viral populations. We then subjected viral cDNA from these populations to long-read nanopore sequencing. Remarkably, both populations were substantially enriched for missense mutations in the Rs3367-CoV spike gene that are consistent with our overarching hypothesis that this spike exists in a predominantly closed conformation in its WT state. A majority of the clones in population 1 independently bore mutations encoding substitutions V584I and V946G. A majority of the clones in population 2 were double mutants with Q836P and N943H substitutions. As described in the revised manuscript, these substitutions cluster either in the upper β-sheet of the CTD2 and interact with Y623 (V584I) or in the FPPR (Q836P, N943H, V946G), which we have already identified as part of a ‘rheostat’ that influences spike opening (Tse, PLoS Pathogens, 2024). Given the bottlenecked nature of VSV reverse genetics, it is not unexpected that we did not obtain Y623H in these experiments. Nonetheless, the fact that multiple rescue attempts with VSVs encoding different Clade Ia bat sarbecovirus spikes have yielded related—but distinct—amino acid changes in the same equatorial region of the spike trimer suggests that many other substitutions that afford spike opening are possible. These new findings are now reported in **Fig. 6**, **S9 Fig.,** and **S3 File**.

Treatment of sarbecovirus spike proteins with trypsin frequently augments infectivity but can also allow for ACE2-independent entry. Was entry driven by trypsin-treated WIV-1 S and Rs3367-S ACE2-dependent?

We have conducted new experiments to assess the effect of trypsin treatment on viral ACE2 dependence (see below). Specifically, we now show that, although trypsin treatment augments entry in ACE2-expressing cells, it does not do so in ACE2-null cells. Thus, we find no evidence for ACE2-independent entry with these particular spikes under these experimental conditions.

Reviewer #3: 1. In this VSV-based pseudovirus infection system with ACE2-expressing DBT-9 cells, does virus entry occur via the cell surface pathway, the endocytic pathway, or both? Does the Y623H substitution drive preferential utilization of one pathway compared to the other? This may be key to understanding the mechanism of altered entry and is brought up briefly in discussion but not investigated. Experimental determination of this would contribute greatly to study novelty and impact relative to previously published work.

We show that entry into ACE2-expressing cells by both Rs3367-CoV WT and Y623H spikes is sensitive to a lysosomotropic compound, NH_4_Cl (**S10 Fig.**). These findings indicate that both spikes depend on the endosomal route and that the Y623H substitution does not drive a change in the viral preference for the route of entry. Rather, they reinforce our hypothesis that Y623H opens the spike, allowing the Rs3367-CoV spike to engage ACE2 at the cell surface prior to its internalization into endosomes and viral membrane fusion.

2. While the manuscript clearly demonstrates that spike variants do not differ in expression in plasmid-transfected cells, what about incorporation of spike into VSV particles? It would be more convincing to test the level of spike present in purified virions, such as Western blot on sucrose gradient-purified pseudovirus (standardized by genome equivalents).

Please see our responses to Reviewer 1. Spike incorporation into viral particles standardized by genome equivalents is now reported in the revised manuscript (**S1E–F Fig.**).

3. Conclusions are made regarding adaptation of a Rs3367-like CoV to human and primate (Vero E6) cell culture, but cell entry is tested using only bat host (Rhinolophus affinis) ACE2. Would results differ if human or primate ACE2 were tested? Why test RaACE2 for entry and human ACE2 for binding? Could amino acid differences between ACE2 derived from these species affect the results?

Previous studies have shown that the isolated WIV1-CoV RBD (identical to that of Rs3367-CoV) binds with nanomolar affinity to diverse ACE2 orthologs, including from human, civet, pangolin, mouse, intermediate horseshoe bat (R. affinis), and Chinese rufous horseshoe bat (R. sinicus) (Starr et al., 2022). Indeed, an extensive body of published data points to the broad ACE2 receptor use by Clade Ia sarbecoviruses (Lee et al., 2023). Therefore, the modest differences in ACE2 ortholog-dependent binding affinity are unlikely to substantially influence our results. Indeed, our new findings that adaptive mutations in the Rs3367-CoV spike (selected during rVSV passage on Vero primate cells) occur outside the RBD and in the sequences that influence spike conformation (see response to Reviewer 1, **Fig. 6**, **S9 Fig,** and **S3 File**) reinforces our overarching conclusion that spike-opening mutations—and not RBD affinity altering/enhancing mutations— are primarily involved in the adaptation of an Rs3367-like virus to cell culture. Nevertheless, the revised manuscript now includes viral entry experiments with VSVs bearing Rs3367-CoV and WIV1-CoV spikes in cells over-expressing human ACE2 (**S10 Fig.**), which illustrate that the effect of the Y623H substitution is independent of the source of ACE2 (human vs. *R. affinis*).
